# Arthroscopic washout of the ankle for septic arthritis in a three-month-old boy

**DOI:** 10.1186/1758-2555-3-21

**Published:** 2011-10-01

**Authors:** Tetsuo Hagino, Masanori Wako, Satoshi Ochiai

**Affiliations:** 1 The Sports Medicine and Knee Center, Department of Orthopaedic Surgery, Kofu National Hospital, 11-35 Tenjin-cho, Kofu, Yamanashi 400-8533, Japan; 2 Department of Orthopaedic Surgery, Faculty of Medicine, University of Yamanashi, Yamanashi, Japan

## Abstract

There is no report of athroscopic treatment for septic arthritis of the ankle in infants. We report a case of successful management of septic arthritis of the ankle in a three-month-old boy by arthroscopic washout. Arthroscopic washout may be a useful treatment for septic arthritis in young infants when performed early after onset.

## Background

Septic arthritis of the ankle is relatively rare, constituting 10 to 15% of all septic arthritis cases in adults [1], and 4 to 13% of cases in children [2-4]. Like septic arthritis of other sites, the important factors influencing the prognosis of septic arthritis of the ankle include early treatment after onset, administration of appropriate antibiotics, and adequate sterilization of the joint. Especially in infants, treatment delay or inappropriate treatment may result in cartilage destruction, avascular necrosis and physeal injury [5,6], with an increased risk of severe deformation and functional impairment. In the past, septic arthritis was managed by repeated needle aspirations or arthrotomy. Recently, arthroscopic treatment has become widely used, both in adults and children [7-11]. Several studies that compared the treatment results of arthroscopic surgery and open surgery for septic arthritis confirmed that arthroscopic procedure is an effective method in treating isolated and uncomplicated arthritic joints, resulting in shorter hospital stay and fewer operations [12-14]. Arthroscopic procedure is especially valuable due to the less invasiveness to the articular cartilage and epiphyses. However, there is no report of arthroscopic treatment for septic arthritis of the ankle in infants. In this report, we describe a case of successful management of septic arthritis of the ankle in an infant by arthroscopic washout.

## Case presentation

A 3-month-old male in-patient was referred to our department from the pediatric department for investigation of suspected septic arthritis of the left ankle.

Three days before referral, the patient developed fever and slight swelling of the left ankle during the night. The next day, he was not able to move the left ankle, and was brought to the emergency outpatient of the pediatric department in our hospital. He had a past history of bronchiolitis 10 days before onset of ankle symptoms, and was treated with oral Rokitamycin (140 mg/day) for one week, resulting in resolution of coughing and other respiratory symptoms. He had no history of injection in the left leg and trauma. There were also no factors related to immunodeficiency. At the initial examination by the pediatrician, his body temperature was 37.6°C. Slight swelling and heat were found in the ankle, but a plain radiograph showed no abnormality. Blood tests showed a white blood cell count of 14,330/mm^3 ^and C-reactive protein of 2.6 mg/l. From the local and systemic findings, the pediatrician suspected septic arthritis. The patient was hospitalized for observation. Splint fixation of the left ankle, cooling, and intravenous infusion of first generation cephalosporin (600 mg/day) were started. However, swelling and heat deteriorated. On the second hospital day, the patient was referred to our orthopedic department.

**Figure 1:**
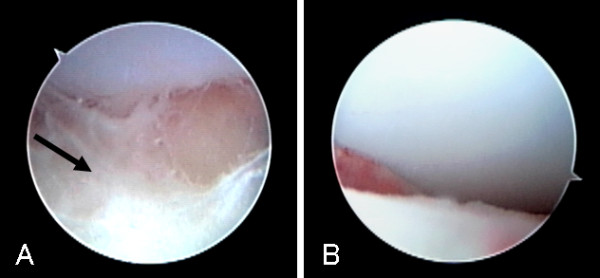
**Arthroscopic findings of the ankle joint in a 3 month-old infant with septic arthritis of the ankle**. A: Arthroscopic findings at the beginning of surgery showed debris (arrow) in the joint space. B: Arthroscopic findings after washout showed no irregularity of the articular cartilage.

**Figure 2:**
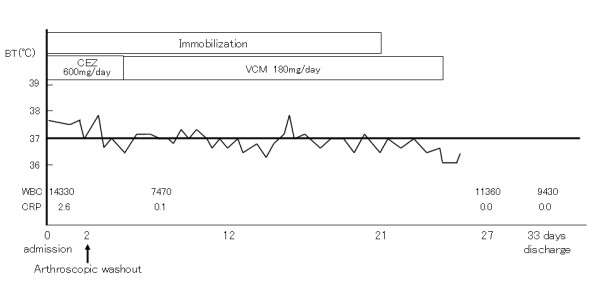
**The clinical course of the present case**. BT: body temperature, CEZ: cephalosporin, VCM: vancomycin, WBC: white blood cell count, CRP: C-reactive protein.

At the first examination by orthopedic surgeons, ultrasonography and magnetic resonance imaging of the left ankle joint showed joint fluid collection. From the symptoms and examination findings, septic arthritis was diagnosed and emergency arthroscopic surgery was conducted. Ultrasonographic examination was performed before operation to confirm the joint gap, anterior tibial tendon and anterior tibial artery. Arthroscopic surgery was conducted in a supine position under general anesthesia. A slight traction was applied to the foot manually. While confirming the position of the joint gap from the image intensifier, a 22-gauge injection needle was inserted from the medial side of the anterior tibial tendon. After confirming that a turbid joint fluid was aspirated, a 5-mm longitudinal incision was made at the same site, and dissected bluntly until reaching inside the joint. Then an arthroscope measuring 1.9 mm in diameter was inserted. After washing with Ringer's solution, a 30° wrist arthroscope was inserted to observe intraarticular changes. While mild hyperplasia of the synovial membrane and debris were observed, irregularity of the articular cartilage was absent (Figure 1). Under arthroscopic visualization, the joint was washed thoroughly until all debris and turbid fluid were removed. A total of 1500 ml of Ringer's solution was used for the washout. Since arthroscopic washout appeared to achieve adequate debridement, no drain was placed after the surgery. The portal was closed with surgical tape. After operation, a splint was applied. One day after surgery, the fever subsided and movement of the left ankle joint became active. Culture of the joint fluid yielded methicillin-resistant *Staphylococcus aureus *(MRSA). Therefore, antibiotic infusion was changed to vancomycin (180 mg/day) on day 2 after surgery. The splint was removed 3 weeks after operation and the patient was discharged on the 31^st ^postoperative day (Figure 2). At the last follow-up six years after the operation, there was no relapse and the patient had no pain and was able to walk without limping.

We obtained consent from the patient's parents to publish this case report.

## Discussion

In general, septic arthritis is managed by needle aspiration as the sole drainage method or by arthrotomy. However, many reports have shown that needle aspiration alone does not achieve adequate clinical result, and several animal studies have verified that needle aspiration is inadequate to manage septic arthritis [15,16]. Forward and Hunter [17] first reported arthroscopic washout as a new technique to treat septic arthritis of the shoulder in infants. Arthroscopic washout was performed under direct arthroscopic vision, with the advantage that complete clearance of pus allowed assessment of the inflammation and the damage to articular cartilage. In addition, if arthroscopic washout alone is not adequate, supplemental treatments can be conducted depending on the arthroscopic findings, such as adding a portal to perform debridement.

Recent reports using a staging system based on arthroscopic findings have proven that the prognosis worsens as the stage advances [10,11]. This evidence supports the necessity of treatment based on intraarticular findings, and that needle aspiration alone may not be sufficient under certain intraarticular conditions.

In the present case, arthroscopy was conducted in the small ankle joint of a 3 month-old infant. Intraarticular observation was possible using an arthroscope 1.9 mm in diameter. The postoperative course has been good cosmetically and functionally. From our experience, arthroscopic washout which is minimally invasive and allows complete washout of the joint is a very useful treatment for septic arthritis of the ankle in infants. However, arthroscopic debridement for treating septic arthritis would be most effective if performed early after onset. Since the risk of articular cartilage damage is increased with the progress of time, in cases where there is a delay in diagnosis and treatment initiation, it is important not to unnecessarily insist on arthrosopic surgery but to consider also open surgery depending on the clinical condition.

## Consent

Written informed consent was obtained from the patient for publication this case report and the images.

## Competing interests

The authors declare that they have no competing interests.

## Authors' contributions

All authors co-wrote the paper and discussed the results for the manuscript preparation. All authors have read and approved the final manuscript.
